# Os Seis Pilares da Medicina do Estilo de Vida no Manejo de Doenças Não Transmissíveis – As Lacunas nas Diretrizes Atuais

**DOI:** 10.36660/abc.20230408

**Published:** 2023-12-19

**Authors:** Rafaella Rogatto de Faria, Sergio Freitas de Siqueira, Francisco Aguerre Haddad, Gustavo Del Monte Silva, Caio Vitale Spaggiari, Martino Martinelli

**Affiliations:** 1 Cultivare Prevenção e Promoção da Saúde Pesquisa e Desenvolvimento São Paulo SP Brasil Cultivare Prevenção e Promoção da Saúde – Pesquisa e Desenvolvimento, São Paulo, SP – Brasil; 2 Hospital das Clínicas da FMUSP Medicina do Esporte São Paulo SP Brasil Medicina do Esporte – Hospital das Clínicas da FMUSP, São Paulo, SP – Brasil; 3 Hospital das Clínicas da FMUSP Instituto do Coração São Paulo SP Brasil Instituto do Coração (InCor), Hospital das Clínicas da FMUSP, São Paulo, SP – Brasil; 4 Pontifícia Universidade Católica de São Paulo São Paulo SP Brasil Pontifícia Universidade Católica de São Paulo, São Paulo, SP – Brasil

**Keywords:** Doenças crônicas não transmissíveis, Estilo de vida, Diretrizes Clínicas, Saúde Holística

## Abstract

**Fundamento:**

As doenças crônicas não transmissíveis (DCNT), também conhecidas como doenças crônicas de longa duração, são consideradas a principal causa de morte e incapacidade em todo o mundo, e os seis pilares da medicina do estilo de vida (nutrição, exercício, controle de tóxicos, manejo do estresse, saúde do sono e conexão social) desempenham um papel importante na gestão holística da sua prevenção e tratamento. Além disso, as diretrizes médicas são os documentos mais aceitos com recomendações para o manejo das DCNT.

**Objetivo:**

O presente estudo tem como objetivo analisar a ausência de pilares de estilo de vida nas principais diretrizes médicas brasileiras sobre as DCNT e identificar evidências na literatura que possam justificar sua inclusão nos documentos.

**Método:**

As diretrizes brasileiras foram selecionadas de acordo com as causas de morte mais relevantes no Brasil, informadas pelo Sistema de Informações sobre Mortalidade publicado pelo Ministério da Saúde em 2019. Os periódicos foram selecionados na biblioteca PUBMED de acordo com a doença e os pilares do estilo de vida não mencionados.

**Resultados:**

Causas relevantes de mortes no Brasil são o infarto agudo do miocárdio (IAM), o diabetes mellitus (DM) e as doenças pulmonares obstrutivas crônicas (DPOC). Foram identificadas seis diretrizes relacionadas a essas DCNT e todas abordam aspectos do estilo de vida, mas apenas uma, referente à prevenção cardiovascular, destaca todos os seis pilares. Apesar disso, uma pesquisa bibliográfica envolvendo mais de 50 artigos mostrou que há evidências de que todos os pilares podem ajudar no controle de cada uma dessas DCNT.

**Conclusão:**

Raramente os seis pilares do estilo de vida são contemplados nas diretrizes brasileiras para IAM, DM e DPOC. A revisão da literatura identificou evidências de todos os pilares do estilo de vida para oferecer uma abordagem holística para a gestão e prevenção das DCNT.

## Introdução

As doenças crônicas não transmissíveis (DCNT) são definidas como condições crônicas que não resultam de um processo infeccioso (agudo) e, portanto, são “não transmissíveis”, com efeitos persistentes que podem ter impacto nas atividades diárias e exigir atenção médica contínua.^1 , 2^

**Figure:**
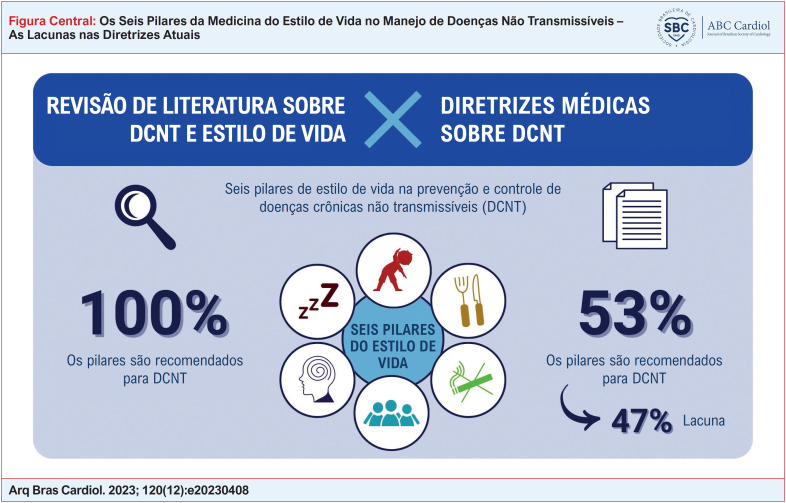


Segundo a Organização Mundial de Saúde (OMS), as DCNT são consideradas a principal causa de morte e incapacidade em todo o mundo, sendo responsáveis por 71% de todas elas.^2^ No Brasil, elas correspondem a 72% de todas as causas de morte.^3^

As DCNT trazem consequências não apenas para os pacientes, mas também para a comunidade em que estão inseridos.^4 , 5^ Assim, a estratégia para enfrentar as DCNT deve incluir uma abordagem holística.^6^

O American College of Lifestyle Medicine define “medicina do estilo de vida” como “o uso de intervenção terapêutica no estilo de vida baseada em evidências como modalidade primária, para prevenir, tratar e muitas vezes reverter doenças crônicas”.^7^

A base da prática da medicina do estilo de vida envolve seis pilares: alimentação saudável, atividade física regular, cessação do tabagismo e controle de substâncias tóxicas, estratégias para controlar o estresse, melhoria do sono e conexões sociais.^8^

O estilo de vida, além de não ser invasivo e ter custo relativamente baixo, afeta positivamente os aspectos fisiológicos, metabólicos, psicológicos e sociais. Mudanças no estilo de vida poderiam beneficiar a complacência pulmonar, a resistência cardíaca, a oxigenação cerebral, a disposição e o desempenho, a imunidade, o processo de aprendizagem, entre outros aspectos que contribuem para a redução da incidência de DCNT.^5 , 9 - 15^ Portanto, as abordagens do estilo de vida desempenham um papel importante na gestão holística da prevenção e do tratamento das DCNT.

Por outro lado, as diretrizes médicas são os documentos mais aceitos com recomendações para o manejo das DCNT. As diretrizes são reconhecidas por conterem as melhores evidências científicas disponíveis sobre o assunto e também podem considerar a análise da relação custo-benefício de uma conduta clínica.^16 , 17^

Dessa forma, o presente estudo tem como objetivo analisar a ausência de pilares de estilo de vida nas principais diretrizes médicas brasileiras sobre as DCNT e identificar evidências na literatura que possam justificar sua inclusão nos documentos.

## Métodos

Esta é uma revisão das diretrizes brasileiras sobre DCNT com o objetivo de verificar quais dos seis pilares do estilo de vida não são abordados no documento e encontrar evidências na literatura que possam justificar a inclusão de novas recomendações de mudanças no estilo de vida para melhor manejo dessas doenças.

A seleção das diretrizes médicas brasileiras foi feita de acordo com as causas relevantes de morte no Brasil, apresentadas pelo Sistema de Informações sobre Mortalidade (SIM) divulgado pelo Ministério da Saúde, que informa a taxa de mortalidade por categoria da Classificação Internacional de Doenças 10 (CID 10).^18^

Os periódicos foram selecionados na biblioteca PUBMED por título e resumo, de acordo com a doença e os pilares do estilo de vida não mencionados, registrando os mecanismos de ação e benefícios para o manejo das DCNT ( Material suplementar ).

## Resultados

O SIM de 2022 relata dados de 2019. As causas relevantes de mortes no Brasil estão listadas na Tabela 1 .^18^ Como a pneumonia causada por um microrganismo não é considerada uma doença crônica, as DCNT consideradas para análise foram infarto agudo do miocárdio (IAM), diabetes mellitus (DM) e doenças pulmonares obstrutivas crônicas (DPOC). O IAM é um evento agudo causado principalmente pela condição crônica conhecida como doença cardíaca coronária que causa bloqueio do fluxo sanguíneo, e por esse motivo o IAM foi incluído no estudo.

**Tabela 1:** Categorias da Classificação Internacional de Doenças 10 (CID 10) e número de mortes no Brasil, em 2019

CID-10	Categoria do CID-10	Número de mortes
I21	Infarto agudo do miocárdio	95.557
J18	Pneumonia por microrganismo não especificado	64.651
E14	Diabetes mellitus não especificado	50.238
J44	Outras doenças pulmonares obstrutivas crônicas	41.922
X95	Agressão por disparo de outra arma de fogo ou arma não especificada	27.916
I64	Acidente vascular cerebral, não especificado como hemorrágico ou isquêmico	33.895
C34	Neoplasia maligna de brônquios e pulmões	29.254
I50	Insuficiência cardíaca (ou falência cardíaca)	27.080
I10	Hipertensão essencial	26.560
I11	Doença cardíaca hipertensiva	19.879

Fonte: Sistema de informações sobre mortalidade (SIM) consultado em junho de 2022.^18^

CID: Classificação Internacional de Doenças.

Foram identificadas seis diretrizes abordando as três DCNT com maior taxa de mortalidade no Brasil considerando a categoria CID-10, incluindo uma diretriz genérica sobre prevenção de doenças cardiovasculares e uma relacionada à doença coronariana crônica, uma das causas do IAM.

- Diretriz de Doença Coronária Estável da Sociedade Brasileira de Cardiologia de 2014^19^
- V Diretriz da Sociedade Brasileira de Cardiologia sobre tratamento do infarto agudo do miocárdio com supradesnível do segmento ST de 2015^20^
- Diretriz de Prevenção Cardiovascular da Sociedade Brasileira de Cardiologia 2019^21^
- Diretrizes da Sociedade Brasileira de Cardiologia sobre angina instável e infarto agudo do miocárdio sem supradesnível do segmento ST de 2021^22^
- Protocolo Clínico e Diretrizes Terapêuticas da Doença Pulmonar Obstrutiva Crônica, do Ministério da Saúde, de 2021^23^
- Diretriz Oficial da Sociedade Brasileira de Diabetes de 2022^24^

A Tabela 2 resume quais pilares de estilo de vida foram encontrados em cada uma das diretrizes selecionadas.

**Tabela 2:** Pilares de estilo de vida apresentados nas diretrizes médicas estudadas

Doença da diretriz	Ano da diretriz	Atividade física	Dieta	Controle de tóxicos	Saúde mental	Qualidade do sono	Conexões sociais
DCAE^19^	2014	Sim	Sim	Não	Não	Não	Não
IAM^20^	2015	Sim	Sim	Sim	Não	Não	Não
DCV^21^	2019	Sim	Sim	Sim	Sim	Sim	Sim
IAM^22^	2021	Sim	Sim	Sim	Não	Não	Não
DPOC^23^	2021	Não	Não	Sim	Não	Não	Não
DM^24^	2022	Sim	Sim	Não	Sim	Não	Sim

Fonte: elaborada pelos autores.

DCAE: doença coronária arterial estável; IAM: infarto agudo do miocárdio; DCV: doença cardiovascular; DPOC: doença pulmonar obstrutiva crônica; DM: diabetes mellitus.

### Infarto agudo do miocárdio

#### Quais são as lacunas?

As diretrizes do IAM não mencionam qualidade do sono, saúde mental e conexões sociais. Quando se trata de controle de tóxicos, apenas a cessação do tabagismo foi incluída nas diretrizes do IAM, mas não na Diretriz de doença coronária estável. A redução do consumo de álcool não foi mencionada em nenhuma delas.

Em relação ao pilar de saúde do sono, conforme estudo descritivo de Andrechuck e Ceolim (2015), três alterações prevalecem e afetam o processo de recuperação de pacientes com IAM: má qualidade do sono, sono diurno excessivo e alto risco de síndrome da apneia obstrutiva do sono.^25^ Vários estudos epidemiológicos envolvendo gêneros mistos, números de pacientes diferentes e períodos de acompanhamento diferentes foram realizados para demonstrar a relação mencionada acima.^26 - 32^

Lao et al.^33^ (2018), em estudo de coorte prospectivo com 60.586 adultos e com duração de 18 anos, demonstraram que a má qualidade e a duração inadequada do sono estão associadas a maior risco de doenças cardiovasculares (DCV). Ayas et al.^29^ (2003), acompanhando 71.617 mulheres adultas durante um estudo de 10 anos, mostraram que sono curto (≤ 5 horas) e longo (≥ 9 horas) estão relacionados a um risco aumentado de doença cardíaca coronária.

Além disso, a combinação dos quatro pilares – atividade física regular, dieta equilibrada, controle de substâncias tóxicas e qualidade do sono – demonstrou reduzir o risco de DCV fatais e não fatais, incluindo infarto do miocárdio. Isso foi testado por meio de estudo de coorte prospectivo, conhecido como MORGEN, que acompanhou 8.128 homens e 9.759 mulheres por um período de 10 a 14 anos. Em uma única análise dos pilares, ajustada por idade, sexo e escolaridade, a duração suficiente do sono (≥ 6 horas) apresentou uma relação de risco protetora de 0,76 (intervalo de confiança de 95% - IC 0,63 – 0,91) para DCV composta e, 0,55 (IC 95% 0,38 – 0,80) para DCV fatal.^34^

Os distúrbios de saúde mental estão associados a um maior risco de DCV e ao aumento da mortalidade cardiovascular. Um estudo de coorte com 880 pacientes demonstrou que os transtornos de saúde mental podem ser considerados uma das causas do desenvolvimento da doença, bem como um agravante desta, principalmente quando se trata de IAM.^35^ Além disso, no estudo INTERHEART com quase 30 mil participantes de 52 países, os fatores psicossociais foram considerados um dos três mais importantes fatores de risco associados ao infarto do miocárdio.^36^ Portanto, intervenções focadas nesse pilar são essenciais para reduzir a prevalência de DCV.^37^

Relacionamento social é outro pilar que pode influenciar antes e depois do aparecimento de uma DCV. Numa revisão sistemática e meta-análise de estudos observacionais longitudinais, a solidão e o isolamento social foram associados a um maior risco de doença cardíaca coronária e acidente vascular cerebral.^38 , 39^ Além disso, o aumento do risco de morbidade e mortalidade após sofrer um IAM está ligado ao apoio social, demonstrando como esse pilar afeta os resultados clínicos das DCV.^40^

Em relação ao consumo de álcool, Biddinger et al.^41^ (2022) utilizando dados do Biobank do Reino Unido, de 371.463 indivíduos não aparentados de ascendência genética europeia, concluíram que os conhecidos efeitos protetores cardiovasculares da ingestão leve a moderada de álcool estão relacionados a uma melhor saúde autorrelatada, envolvendo menores taxas de tabagismo e índice de massa corporal, e maior atividade física e ingestão de vegetais. Em contraste, o consumo excessivo de álcool foi associado a um maior aumento nos riscos de DCV.

Como complemento a este estudo, uma revisão sistemática com 23 estudos observacionais incluindo 29.457 participantes também mostrou que o consumo excessivo de álcool pode provocar um risco cardiovascular imediato e contínuo, após as primeiras 24 horas de ingestão. Por outro lado, o consumo moderado foi associado a maior risco cardiovascular imediatamente após a ingestão e antes de 24 horas após a ingestão.^42^

### Diabetes mellitus

#### Quais são as lacunas?

As deficiências observadas na diretriz de DM são a qualidade do sono e o controle de substâncias tóxicas, como a cessação do tabagismo.

Os distúrbios do sono são considerados fator de risco e agravantes da doença. Uma revisão sistemática e meta-análise sem estudos prospectivos randomizados envolvendo 22 estudos com 69.329 participantes mostraram que a má qualidade do sono e a curta duração do sono são riscos independentes para um pior controle glicêmico.^43^ Dois estudos transversais autorrelatados com 16.893 chineses^44^ e 300 pacientes^45^ mostraram que o DM II está associado à má qualidade do sono e à curta duração do sono.

Além disso, evidências laboratoriais mostram que a perda crônica de sono parece estar associada a alterações neuroendócrinas e metabólicas que aumentam o risco de DM II. Em estudo de revisão, van Cauter et al.^46^ (2007) observaram que essas alterações afetam o apetite, com níveis mais baixos de leptina e mais altos de grelina, e causam distúrbios glicêmicos, com menor responsividade celular e sensibilidade à insulina. A hipersecreção de leptina, hormônio que aumenta a ingestão alimentar, pode levar à obesidade, condição predisponente ao DM II.^47^

Nilsson et al.^48^ (2004) estudando uma coorte de 6.599 homens suecos de meia-idade, não diabéticos, observaram, em um acompanhamento médio de 14,8 anos, que a privação de sono aumenta o risco de DM tipo II em 52% (razão de chance 1,52 [IC 95% 1,05 – 2,20]).

Em relação aos controles de tóxicos, de acordo com os Centros de Controle e Prevenção de Doenças (CDC),^49^ o risco de fumantes desenvolverem DM II é de 30 a 40 por cento maior do que o de não fumantes. Isso se deve a alterações na funcionalidade do corpo e danos celulares causados pelos produtos químicos, levando ao stress oxidativo e à inflamação, diminuindo a eficácia da insulina.^49 - 52^

Quando se trata do consumo de álcool, apesar de ser um assunto polêmico, uma revisão sistemática e meta-análise incluindo 20 estudos de coorte confirmaram a relação em forma de U com o risco de DM II, em que o consumo moderado apresenta fator de proteção.^53^ Além disso, um estudo prospectivo com 5.521 homens com idade entre 40 e 59 anos mostrou que o consumo excessivo de álcool, mediado pelo peso corporal, está associado à incidência de DM II.^54^

### Doenças pulmonares obstrutivas crônicas

#### Quais são as lacunas?

A diretriz para DPOC não abordou atividade física, dieta, saúde mental, qualidade do sono e conexões sociais.

A saúde mental tem impacto direto nas DPOC e pode ser causa ou consequência da doença. Uma revisão sistemática e uma meta-análise foram realizadas utilizando 16 estudos sobre depressão ou ansiedade como preditores de risco ou mortalidade por DPOC envolvendo 28.759 indivíduos, e 6 estudos sobre doenças relacionadas à DPOC como preditores de depressão envolvendo 7.439.159 indivíduos. Esse estudo apontou que a ansiedade e a depressão podem afetar negativamente o prognóstico da DPOC, podendo aumentar o risco de hospitalizações e exacerbações.^55^

Montserrat-Capdevila et al.^56^ (2018), em estudo de coorte prospectivo com 512 pacientes com DPOC originários de uma área rural da Espanha, acompanhados entre 2012 e 2014, mostraram que o diagnóstico de ansiedade e depressão quase dobrou o risco de hospitalização por exacerbação grave da DPOC.

Gudmundsson et al.^57^ (2005), em estudo prospectivo multicêntrico envolvendo 416 pacientes nos países nórdicos, mostraram maior prevalência de ansiedade e depressão em pacientes que receberam alta após hospitalização por exacerbação aguda da DPOC.

No que diz respeito à nutrição, existem algumas opções alimentares a seguir para melhorar a saúde respiratória, especificamente na prevenção da DPOC, todas derivadas de estudos clínicos e observacionais, como a dieta mediterrânea.^58^

Um estudo transversal com 207 fumantes mostrou a associação entre os efeitos adversos do consumo de álcool e a dieta ocidental, rica em alimentos refinados, gordura saturada, carne e açúcar, e o comprometimento da função pulmonar. Em oposição, a dieta mediterrânea, repleta de alimentos vegetais e gorduras saudáveis, parece preservar a função pulmonar e prevenir a DPOC ou a sua progressão.^58^

Evidências epidemiológicas coletadas de 25 artigos sugerem um efeito positivo da ingestão de frutas, peixes e vegetais, incluindo benefícios para a função pulmonar e uma relação inversa com a mortalidade por DPOC e sintomas respiratórios. O oposto é observado em altos níveis de consumo de carne.^59^ Além disso, um estudo caso-controle com 183 idosos, incluindo 21 indivíduos com DPOC, mostrou que pacientes com DPOC apresentavam dieta de pior qualidade antioxidante.^60^ A dieta antioxidante foi previamente associada a benefícios para a saúde pulmonar em um estudo transversal com 14.120 adultos.^61^

Estudos que associam atividade física e DPOC estão relacionados aos desfechos que os exercícios podem trazer para quem já tem a doença. É um fator importante para a reabilitação pulmonar, exigindo consideração de duração, intensidade e modos de atividade.

Em todo o mundo, as diretrizes de prática clínica para o manejo da DPOC foram revisadas por Lewthwaite et al.^62^ (2017) de 2005 a 2017. Eles encontraram vinte e um documentos recomendando atividade física para melhorar os resultados de saúde dos pacientes com DPOC.

Donaire-Gonzalez et al.^63^ (2015), em estudo prospectivo envolvendo 177 pacientes com acompanhamento de 2 anos, demonstraram que maior quantidade de exercício físico de baixa intensidade reduz o risco de hospitalizações por DPOC. Garcia-Aymerich et al.^64^ (2006), em estudo de coorte prospectivo com 28.747 pessoas ao longo de 12 anos de acompanhamento, acrescentam que atividade física baixa, moderada e alta proporciona menor risco de hospitalização por DPOC.

De acordo com Watz et al.^65^ (2008) e Waschki et al.^66^ (2011) em estudo prospectivo com 170 pacientes com DPOC estável, a inatividade física ou o sedentarismo podem levar à diminuição da função pulmonar e cardíaca, inflamação sistêmica e fraqueza muscular, que afetam os desfechos clínicos, além de aumentar o risco de mortalidade. Dogra et al.^67^ (2018) em um estudo longitudinal com 877 canadenses com DPOC mostraram os efeitos negativos do tempo sedentário na percepção da saúde, na saúde mental e no envelhecimento.

Quanto ao pilar de apoio social em pacientes com DPOC, a maioria dos estudos associou a relação do apoio social com aspectos de saúde mental. DiNicola et al.^68^ (2013) e Marino et al.^69^ (2008), em estudos transversais com 452 e 156 pessoas, respectivamente, avaliaram a importância do apoio social percebido para pacientes com DPOC, quando estes apresentam ansiedade e depressão como comorbidades. DiNicola et al.^68^ (2013) afirmaram que o apoio social positivo e negativo foram preditores significativos de ansiedade em pacientes com DPOC. Marino et al.^69^ (2008) demonstraram que o apoio social e a autoeficácia estavam relacionados ao funcionamento social geral.

Em uma revisão de escopo incluindo 31 estudos, Barton et al.^70^ (2015) descreveram que o apoio social adequado foi benéfico para o autocuidado e adesão ao tratamento em pacientes com DPOC, além de trazer desfecho positivo em relação à saúde mental.

Um estudo de revisão e outro estudo realizado com 24 pacientes com DPOC associando a qualidade do sono à DPOC demonstram o impacto da doença na rotina de sono e como ela pode exacerbar os efeitos da doença. Distúrbios do sono, como insônia, são comuns em pacientes com DPOC. A dessaturação de oxigênio noturna ocorre mesmo na DPOC leve e pode refletir distúrbios respiratórios do sono ou hipoventilação relacionada ao sono com movimento rápido dos olhos.^71 , 72^

Vukoja et al.^73^ (2018) em estudo transversal com 100 pacientes com DPOC e 104 indivíduos saudáveis demonstraram que aqueles com a doença apresentavam qualidade de sono ruim, sendo significativamente superior em comparação ao grupo de controle. Serin et al.^74^ (2020) em estudo com 110 pacientes com DPOC concluíram que esses indivíduos apresentavam qualidade de sono moderada ou ruim e dispneia.

Omachi et al.^75^ (2012) em um estudo envolvendo 98 adultos mostraram que, em uma investigação transversal, o sono perturbado estava associado à piora da DPOC, além de indicar que, em uma análise longitudinal, esse sono perturbado estava associado a exacerbações, utilização de serviços de saúde de emergência e mortalidade. De acordo com os estudos de Budhiraja et al.^76^ (2015) e Greenberg e Goss^77^ (2009), o manejo ideal dessas doenças requer tratamento para ambas as condições para melhorar os resultados globais, incluindo farmacológicos e não farmacológicos, como cessação do tabagismo, suplementação de oxigênio, educação sobre higiene do sono e outros.

## Discussão

Nossos resultados mostram que todas as orientações médicas abordam alguns aspectos do estilo de vida, mas o único documento que destaca todos os seis pilares foi o documento de 2019 sobre prevenção contra doenças cardiovasculares. Apesar disso, uma pesquisa bibliográfica mostrou que há evidências de que todos os pilares podem auxiliar no controle das DCNT, embora eles não tenham sido mencionados nas diretrizes. Os pilares mais comuns encontrados foram atividade física, nutrição e controle de substâncias tóxicas.

O impacto das mudanças no estilo de vida sobre as DCNT é difícil de avaliar em ensaios clínicos randomizados por diversas razões, incluindo aspectos éticos. Fatores como privação de sono ou incentivo ao uso de drogas ou ao consumo de bebida alcoólica são antiéticos, por exemplo. Por essas razões, a maior parte das evidências disponíveis na literatura e apresentadas nos nossos resultados baseia-se em estudos epidemiológicos.^25 - 36 , 41 , 44 , 45 , 48 , 52 , 54 , 56 - 61 , 65 - 69 , 73 - 75^

Uma comparação direta entre as diretrizes clínicas e as recomendações de estilo de vida só foi realizada anteriormente por Lewthwaite et al.^62^ (2017) em um estudo de revisão sistemática associando atividade física e comportamentos de sono nas diretrizes para DPOC. Assim, isso aumenta a importância do nosso estudo ao incluir todos os pilares do estilo de vida, ao mesmo tempo em que aborda mais de uma DCNT.

Diretrizes médicas são documentos que contêm as melhores evidências científicas disponíveis sobre um tema, utilizados para melhorar a qualidade do atendimento aos pacientes e melhorar a eficácia clínica.^78^ Porém, a maioria das diretrizes atualmente se concentra no processo de tratamento, principalmente farmacológico, descartando abordagens importantes relacionadas ao estilo de vida e aos mecanismos preventivos.

No entanto, nota-se que as diretrizes têm incorporado cada vez mais aspectos de estilo de vida às suas recomendações. Nas Diretrizes da Sociedade Brasileira de Diabetes de 2019-2020, os únicos pilares mencionados foram atividade física e dieta.^79^ Entretanto, em 2022, foram incorporados aspectos psicossociais, que são extremamente relevantes para o paciente.^24^ Esses mesmos pilares de estilo de vida foram acrescentados às diretrizes publicadas mais recentemente sobre hipertensão, tanto como ferramentas preventivas quanto de tratamento.^80^ Mais recentemente, no final de 2022, após o encerramento do presente estudo, foi publicado um posicionamento sobre Saúde Cardiovascular da Mulher com recomendações de aspectos relacionados a todos os pilares do estilo de vida como medida preventiva de doenças cardiovasculares.^81^ Espera-se que isso seja uma tendência e, portanto, uma boa atitude para que outras diretrizes iniciem a incorporação de outros pilares, como foi feito na Diretriz de Prevenção Cardiovascular 2019, que enquadra as doenças cardiovasculares de forma genérica.

### Limitações

Dentre as limitações, os estudos reunidos para análise, que servem de base para demonstrar os efeitos dos pilares do estilo de vida no manejo da doença, não são necessariamente ensaios clínicos randomizados principalmente pelas razões éticas discutidas anteriormente. A pesquisa por artigos relacionados ao estilo de vida e aos assuntos das diretrizes estudadas não atendeu aos rigores de uma revisão sistemática, pois não era o objetivo deste artigo.

Além disso, este estudo focou nas diretrizes médicas brasileiras, e não ampliou a análise e a comparação às diretrizes americanas ou europeias. Embora as DCNT sejam um problema mundial, a esfera nacional foi escolhida primeiro para aumentar a conscientização.

### Passos futuros

Ao estudar o estilo de vida e seu impacto nos processos de saúde, pouco se fala sobre aspectos ambientais e socioeconômicos. A Diretriz de Prevenção Cardiovascular publicada em 2019, abordada neste estudo, apresenta uma seção sobre o impacto desses fatores nos cuidados de saúde, sendo essa uma nova linha de estudo a ser explorada que pode até se enquadrar como um futuro pilar de estilo de vida não relacionado a medidas comportamentais.

Além disso, outra forma de aprimorar o estudo é informar sobre as DCNT que mais causam mortes no Brasil, não considerando apenas a categoria da CID-10 informada pelo SIM.

## Conclusão

Raramente os seis pilares do estilo de vida são contemplados nas diretrizes brasileiras para IAM, DM e DPOC. A revisão da literatura identificou evidências de todos os pilares do estilo de vida para oferecer uma abordagem holística para a gestão e prevenção dessas DCNT.

## *Material suplementar

Para informação adicional, por favor, clique aqui .
