# Supplementary material for: Os Seis Pilares da Medicina do Estilo de Vida no Manejo de Doenças Não Transmissíveis – As Lacunas nas Diretrizes Atuais
Source: Arq Bras Cardiol. 2023 Dec 19;120(12):e20230408. [Article in Portuguese] doi: 10.36660/abc.20230408 (PMC10735241; doi:10.36660/abc.20230408)
Supplement: Translation

# Translation: The Six Pillars of Lifestyle Medicine in Managing Noncommunicable Diseases – The Gaps in Current Guidelines

**Authors:** Rafaella Rogatto de Faria, Sergio Freitas de Siqueira, Francisco Aguerre Haddad, Gustavo Del Monte Silva, Caio Vitale Spaggiari, Martino Martinelli

**Affiliations:** 1 São Paulo SP Brazil Cultivare Prevenção e Promoção da Saúde – Pesquisa e Desenvolvimento, São Paulo, SP – Brazil; 2 São Paulo SP Brazil Medicina do Esporte – Hospital das Clínicas da FMUSP, São Paulo, SP – Brazil; 3 São Paulo SP Brazil Instituto do Coração (InCor), Hospital das Clínicas da FMUSP, São Paulo, SP – Brazil; 4 São Paulo SP Brazil Pontifícia Universidade Católica de São Paulo, São Paulo, SP – Brazil

**Keywords:** Noncommunicable Diseases, Life Style, Practice Guideline, Holistic Health

## Abstract

**Background:**

Noncommunicable diseases (NCDs), also known as chronic diseases that are long-lasting, are considered the major cause of death and disability worldwide, and the six pillars of lifestyle medicine (nutrition, exercise, toxic control, stress management, restorative sleep, and social connection) play an important role in a holistic management of their prevention and treatment. In addition, medical guidelines are the most accepted documents with recommendations to manage NCDs.

**Objective:**

The present study aims to analyze the lack of lifestyle pillars concerning the major Brazilian medical guidelines for NCDs and identify evidence in the literature that could justify their inclusion in the documents.

**Method:**

Brazilian guidelines were selected according to the most relevant causes of death in Brazil, given by the Mortality Information System, published by the Brazilian Ministry of Health in 2019. Journals were screened in the PUBMED library according to the disease and non-mentioned pillars of lifestyle.

**Results:**

Relevant causes of deaths in Brazil are acute myocardial infarction (AMI), diabetes mellitus (DM), and chronic obstructive pulmonary diseases (COPD). Six guidelines related to these NCDs were identified, and all address aspects of lifestyle, but only one, regarding cardiovascular prevention, highlights all six pillars. Despite this, a literature search involving over 50 articles showed that there is evidence that all the pillars can help control each of these NCDs.

**Conclusion:**

Rarely are the six pillars of lifestyle contemplated in Brazilian guidelines for AMI, DM, and COPD. The literature review identified evidence of all lifestyle pillars to offer a holistic approach for the management and prevention of NCDs.

## Introduction

Noncommunicable diseases (NCDs) are defined as chronic conditions that do not result from an (acute) infectious process, and thus are “not communicable”, with persistent effects that can impact daily activities and require ongoing medical attention.^1 , 2^

According to the World Health Organization (WHO), NCDs are considered the major cause of death and disability worldwide, responsible for 71% of all of deaths.^2^In Brazil, it corresponds to 72% of all causes of death.^3^

NCDs have consequences not only for the patients, but also or the community in which they are inserted.^4 , 5^Hence, the strategy to face the NCDs must include a holistic approach.^6^

Meanwhile, the American College of Lifestyle Medicine defines “lifestyle medicine” as *“the use of evidence-based lifestyle therapeutic intervention as a primary modality, to prevent, treat and often reverse chronic disease”.* ^7^

The basis of lifestyle medicine practice involves six pillars: healthy eating, regular physical activity, cessation of tobacco use and toxic control, strategies to manage stress, sleep improvement, and social connections.^8^

Lifestyle, in addition to being non-invasive and having a relatively low cost, positively impacts physiological, metabolic, psychological, and social aspects. Changes in lifestyle could benefit pulmonary compliance, cardiac resistance, brain oxygenation, disposition and performance, immunity, learning process, among other aspects that contribute to reducing the incidence of NCDs.^5 , 9 - 15^Therefore, lifestyle approaches play an important role in a holistic management of NCDs prevention and treatment.

By contrast, medical guidelines are the most accepted documents with recommendations to manage NCDs. The guidelines are well-known for containing the best scientific evidence available on the issue and may also consider the cost-effectiveness analysis for a clinical conduct.^16 , 17^

Therefore, the present study aims to analyze the lack of lifestyle pillars in the major Brazilian medical guidelines for NCDs and identify evidence in the literature that could justify their inclusion in the documents.

## Methods

This is a review of NCD Brazilian guidelines, aiming to verify which of the six pillars of lifestyle are not addressed in the document and find evidence in the literature that could justify the inclusion of new recommendations of lifestyle changes to better manage these diseases.

The selection of the Brazilian medical guidelines was done according to relevant causes of death in Brazil, established by the Mortality Information System (SIM, in Portuguese) published by the Ministry of Health, which informs the mortality rate by International Classification of Diseases 10 (ICD 10) category.^18^

Journals were screened in the PUBMED library by title and abstract according to the disease and the non-mentioned pillars of lifestyle, enrolling the mechanisms of actions and benefits to manage the NCDs ( Supplementary Material ).

## Results

The SIM from 2022 reports data from 2019. Relevant causes of death in Brazil are listed in Table 1 .^18^As pneumonia caused by a microorganism is not considered a chronic disease, the NCDs considered for analysis were acute myocardial infarction (AMI), diabetes mellitus (DM), and chronic obstructive pulmonary diseases (COPD). The AMI is an acute event, mostly caused by a chronic condition, known as coronary heart disease, which causes blockage of blood flow; thus, AMI was included in the study.

**Table 1:** International Classification of Diseases 10 (ICD 10) categories and number of deaths in Brazil, in 2019

ICD-10	ICD-10 Category	Number of Deaths
I21	Acute myocardial infarction	95,557
J18	Pneumonia by microorganism not specified	64,651
E14	Diabetes mellitus not specified	50,238
J44	Other chronic obstructive pulmonary disease	41,922
X95	Assault by firing another firearm or unspecified weapon	27,916
I64	Stroke, not specified as hemorrhagic or ischemic	33,895
C34	Malignant neoplasm of bronchi and lungs	29,254
I50	Cardiac insufficiency (or heart failure)	27,080
I10	Essential hypertension	26,560
I11	Hypertensive heart disease	19,879

Source: Mortality Information System (SIM) consulted in June 2022.^18^

ICD: International Classification of Diseases.

Six guidelines were identified addressing the three NCDs with the highest mortality rate in Brazil considering the ICD-10 category, including a generic category on the prevention of cardiovascular diseases and one related to coronary chronic disease, a cause of AMI.

1. Guidelines of the Brazilian Society of Cardiology on Stable Coronary Disease from 2014^19^
2. Fifth Guideline of the Brazilian Cardiology Society about the treatment of Acute Myocardial Infarction with elevated ST Segment from 2015^20^
3. Guidelines of the Brazilian Society of Cardiology on Cardiovascular Prevention from 2019^21^
4. Guidelines of the Brazilian Society of Cardiology on Unstable Angina and Acute Myocardial Infarction without ST-segment Elevation from 2021^22^
5. Clinical Protocol and Therapeutic Guidelines of Chronic Obstructive Pulmonary Disease, by the Ministry of Health, from 2021^23^
6. Official Guideline of the Brazilian Society of Diabetes from 2022^24^

Table 2 summarizes which lifestyle pillars were found in each of the selected guidelines.

**Table 2:** Lifestyle pillars presented in the medical guidelines studied

Guideline Disease	Guideline Year	Physical Activity	Diet	Toxic Control	Mental Health	Sleep Quality	Social Connections
SCAD^19^	2014	Yes	Yes	No	No	No	No
AMI^20^	2015	Yes	Yes	Yes	No	No	No
CVD^21^	2019	Yes	Yes	Yes	Yes	Yes	Yes
AMI^22^	2021	Yes	Yes	Yes	No	No	No
COPD^23^	2021	No	No	Yes	No	No	No
DM^24^	2022	Yes	Yes	No	Yes	No	Yes

Source: drafted by the authors.

SCAD: stable coronary artery disease; AMI: acute myocardial infarction; CVD: cardiovascular disease; COPD: chronic obstructive pulmonary disease; DM: diabetes mellitus.

### Acute myocardial infarction

#### What are the gaps?

The AMI guidelines do not mention sleep quality, mental health, and social connections. When it comes to toxic control, only the cessation of tobacco use was included in the AMI guidelines, but not in the Stable Coronary Guideline. A reduction in alcohol consumption was not mentioned in any of them.

Regarding the restorative sleep pillar, according to the descriptive study by Andrechuck and Ceolim (2015), three alterations prevail and affect the recovery process of patients with AMI: poor sleep quality, excessive daytime sleep, and high risk for obstructive sleep apnea syndrome.^25^Several epidemiological studies involving mixed genres, different numbers of patients, and follow-up periods were carried out to demonstrate the relation mentioned above.^26 - 32^

Lao et al.^33^(2018), in a prospective cohort study with 60,586 adults lasting 18 years, demonstrated that poor sleep quality as well as inadequate sleep duration are associated with a higher risk of cardiovascular diseases (CVDs). Ayas et al.^29^(2003), following up on 71,617 female adults during a 10-year study showed that short (<=5 hours) and long (>=9 hours) sleep are related to an increased risk of coronary heart disease.

Moreover, the combination of the four pillars – regular physical activity, balanced diet, toxic control, and sleep quality – has proven to reduce the risk of fatal and nonfatal CVDs, including myocardial infarction. This was tested by means of a prospective cohort study, known as MORGEN, following 8,128 men and 9,759 women for 10 to 14 years. In a single analysis of the pillars, adjusted for age, sex, and educational level, sufficient sleep duration (>=6 hours) presented a protective hazard ratio of 0.76 (95% Confidence Interval - CI 0.63 – 0.91) for composite CVD and 0.55 (95% CI 0.38 – 0.80) for fatal CVD.^34^

Mental health disorders are associated with a higher risk of CVDs and with increased cardiovascular mortality. A cohort study with 880 patients demonstrated that mental health disorders can be considered as one of the causes of the development of the disease, as well as an aggravating factor for it, especially when it comes to AMI.^35^Moreover, in the INTERHEART study, conducted with almost 30,000 participants from 52 countries, psychosocial factors were considered one of the three most important risk factors associated with myocardial infarction.^36^Therefore, interventions focused on this pillar are essential to reducing CVD prevalence.^37^

A social relation is the other pillar that can influence both before and after acquiring a CVD. In a systematic review and meta-analysis of longitudinal observational studies, loneliness and social isolation were associated with a higher risk for coronary heart disease and stroke.^38 , 39^In addition, increased risk of morbidity and mortality after acquiring AMI are linked to social support, demonstrating how this pillar impacts the clinical outcomes of CVDs.^40^

Regarding alcohol intake, Biddinger et al.^41^(2022), using data from the United Kingdom's Biobank, including 371,463 unrelated individuals of European genetic descent, concluded that the well-known cardiovascular protective effects of light to moderate alcohol intake are related to a better self-reported health, involving lower smoking rates and body mass index, and higher physical activity and vegetable intake. In contrast, heavy alcohol consumption was associated with a higher increase in CVD risks.

As a complement to this study, a systematic review with 23 observational studies, including 29,457 participants, also showed that heavy alcohol consumption could provoke an immediate and continued cardiovascular risk, after the first 24 hours of intake. On the other hand, moderate consumption was associated with higher cardiovascular risk immediately after drinking, and prior to 24 hours after the intake.^42^

### Diabetes Mellitus

#### What are the gaps?

The shortcomings observed in the DM guideline are sleep quality and toxic control, such as the cessation of tobacco use.

Sleep disorders are considered both a risk factor and an aggravator of the disease. A systematic review and meta-analysis with no randomized prospective studies involving 22 studies with 69,329 participants showed that poor sleep quality and short sleep duration are independent risks for poorer glycemic control.^43^Two self-reported cross-sectional studies with 16,893 Chinese^44^and 300 patients^45^showed that DM II is associated with poor sleep quality and short sleep duration.

Moreover, laboratory evidence shows that chronic sleep loss appears to be associated with neuroendocrine and metabolic alterations, which increase the risk for DM II. In a review study, Van Cauter et al.^46^(2007) observed that these alterations affect the appetite, with lower leptin and higher ghrelin levels, and cause glucose disturbances, with lower cell responsiveness and insulin sensitivity. The hypersecretion of leptin, a hormone that increases food intake, can lead to obesity, a condition predisposed to DM II.^47^

Nilsson et al.^48^(2004), studying a cohort of 6,599 Swedish middle-aged non-diabetic men, observed, in a mean follow-up of 14.8 years, that sleep deprivation increases the risk of DM type II by 52% (odds ratio 1.52 [95% CI 1.05–2.20]).

Regarding toxic controls, according to the Centers for Disease Control and Prevention (CDC),^49^the risk of smokers developing DM II is 30 to 40 percent higher than nonsmokers. This happens due to alterations in the body's functionality and cell damage caused by the chemicals, leading to oxidative stress and inflammation, and decreasing insulin effectiveness.^49 - 52^

When it comes to alcohol consumption, despite being a controversial subject, one systematic review and meta-analysis, including 20 cohort studies, confirmed the U-shaped relationship with DM II risk, in which moderate drinking presents a protective factor.^53^Moreover, a prospective study with 5,521 men, aged 40-59 years, showed that heavy alcohol consumption, mediated by body weight, is associated with an incidence of DM II.^54^

### Chronic obstructive pulmonary diseases

#### What are the gaps?

The COPD guideline did not address physical activity, diet, mental health, sleep quality, and social connections.

Mental health has a direct impact on COPD and can either be a cause or a consequence of the disease. A systematic review and meta-analysis were carried out using 16 studies on depression or anxiety as predictors of COPD risk or mortality, involving 28,759 individuals, and 6 studies on COPD-related conditions as a predictor of depression, involving 7,439,159 individuals. This study pointed out that anxiety and depression can negatively affect the COPD prognosis and may increase the risk of hospitalizations and exacerbations.^55^

Montserrat-Capdevila et al.^56^(2018), in a prospective cohort study conducted with 512 patients with COPD, originating from a rural area in Spain, followed up between 2012 and 2014, showed that the diagnosis of anxiety and depression almost doubled the risk of hospitalization due to severe exacerbation of COPD.

Gudmundsson et al.^57^(2005), in a multicenter prospective study, involving 416 patients, in the Nordic countries, showed a higher prevalence of anxiety and depression in patients discharged after hospitalization due to an acute exacerbation of COPD.

When it comes to nutrition, there are some dietary options to be followed to improve respiratory health, specifically in preventing COPD, all derived from clinical and observational studies, such as the Mediterranean diet.^58^

A cross-sectional study, conducted with 207 smokers, showed the association between the adverse effects of alcohol consumption and the Western diet, which is rich in refined food, saturated fat, meat, and sugar, with impaired lung function. In opposition, the Mediterranean diet, replete with plant-based foods and a healthy fat, seems to preserve the lung function and prevent COPD or its progression.^58^

Epidemiological evidence gathered from 25 articles suggests a positive effect of fruit, fish, and vegetable intake, including benefits for the lung function and an inverse relationship to COPD mortality and respiratory symptoms. The opposite is seen for high levels of meat consumption.^59^In addition, a case-control study with 183 elderly people, including 21 individuals with COPD, showed that patients with COPD had a diet of poorer antioxidant quality.^60^The antioxidant diet was previously associated with lung health benefits in a cross-sectional study of 14,120 adults.^61^

Studies associating physical activity and COPD are related to the outcomes that exercises can bring for those who already have the disease. It is an important factor for pulmonary rehabilitation, requiring one to consider the duration, intensity, and modes of activity.

Worldwide, clinical practice guidelines to manage COPD were reviewed by Lewthwaite et al.^62^(2017) from 2005 to 2017. These authors found twenty-one documents recommending physical activity to improve the COPD patients’ health outcomes.

Donaire-Gonzalez et al.^63^(2015) in a prospective study involving 177 patients with a 2-year follow-up, showed that a greater quantity of low-intensity physical exercise reduces the risk of COPD hospitalizations. Garcia-Aymerich et al.^64^(2006), in a prospective cohort study, conducted with 28,747 people throughout 12 years of follow-up, adds that low, moderate, and high physical activity provides a lower risk for hospitalization due to COPD.

According to Watz et al.^65^(2008) and Waschki et al.^66^(2011), in a prospective study with 170 stable COPD patients, physical inactivity or sedentarism can lead to decreased lung and heart function, systemic inflammation, and muscle weakness, which affect clinical outcomes, in addition to increasing the risk of mortality. Dogra et al.^67^(2018), in a longitudinal study with 877 Canadians with COPD, showed the negative effects of sedentary time on perceived health, mental health, and aging.

Regarding the social support pillar in COPD patients, most of the studies associated the relationship of social support with mental health aspects. DiNicola et al.^68^(2013) and Marino et al.^69^(2008), in cross-sectional studies conducted with 452 and 156 people, respectively, assessed the importance of perceived social support for COPD patients, when they have anxiety and depression as comorbidities. DiNicola et al.^68^(2013) stated that positive and negative social support were significant predictors of anxiety in COPD patients. Marino et al.^69^(2008) demonstrated that social support and self-efficacy were related to overall social functioning.

In a scoping review including 31 studies, Barton et al.^70^(2015) described that adequate social support was beneficial for self-care and adherence to treatment in COPD patients, and led to a positive outcome regarding mental health.

One review study and another carried out with 24 COPD patients associating sleep quality with COPD demonstrates the impact of the disease on one's sleep routine and how it can exacerbate the effects of the disease. Sleep disorders like insomnia are common in patients with COPD. Nocturnal oxygen desaturation occurs even in mild COPD and can reflect sleep-disordered breathing or rapid eye movement sleep-related hypoventilation.^71 , 72^

Vukoja et al.^73^(2018) in a cross-sectional study, conducted with 100 COPD patients and 104 healthy individuals, demonstrated that those with the disease had poor sleep quality, being significantly higher when compared to the control group. Serin et al.^74^(2020) in a study with 110 COPD patients, concluded that these individuals had moderate or poor sleep quality and dyspnea.

Omachi et al.^75^(2012), in a study involving 98 adults, showed that, in a cross-sectional investigation, disturbed sleep was associated with a worsening of COPD and, in a longitudinal analysis, was associated with exacerbations, emergency healthcare use, and mortality. According to the studies from Budhiraja et al.^76^(2015) and Greenberg and Goss^77^(2009), optimal management of these diseases requires treatment for both conditions in order to improve overall outcomes, including pharmacological and non-pharmacological, such as the cessation of tobacco use, oxygen supplementation, education about sleep hygiene, among others.

## Discussion

Our results show that all medical guidelines address some aspects of lifestyle, but the only document that highlights all six pillars was the document from 2019 on cardiovascular prevention. Despite this, a literature search showed that there is evidence that all the pillars can help in NCD control; however, they were not mentioned by the guidelines. The most common pillars found were physical activity, nutrition, and toxic control.

The impact of lifestyle changes on NCDs are difficult to assess in randomized clinical trials for several reasons, including ethical aspects. Factors, such as sleep deprivation or encouraging the use of drugs or alcohol consumption, are unethical, for example. For these reasons, most of the evidence available in the literature and displayed in our results is based on epidemiological studies^25 - 36 , 41 , 44 , 45 , 48 , 52 , 54 , 56 - 61 , 65 - 69 , 73 - 75^.

A direct comparison between clinical guidelines and lifestyle recommendations has only been previously performed by Lewthwaite et al.^62^(2017) in a systematic review study associating physical activity and sleep behaviors in COPD guidelines. Thus, this increases the importance of our study by including all pillars of lifestyle whilst addressing more than one NCD.

Medical guidelines are documents that contain the best scientific evidence available on a topic, used to improve the quality of care for patients and improve clinical effectiveness.^78^However, most of the guidelines nowadays focus on the treatment process, mainly pharmacological ones, discarding important approaches related to lifestyle and preventive mechanisms.

Nonetheless, it is noted that the guidelines have increasingly incorporated lifestyle aspects into their recommendations. In the Guidelines from the Brazilian Diabetes Society from 2019-2020, the only pillars mentioned were physical activity and diet.^79^However, in 2022, psychosocial aspects, which are extremely relevant for the patient, were incorporated.^24^Those same lifestyle pillars were added in the most recent guideline published on hypertension, both as both preventive and treatment tools.^80^More recently, in the end of 2022, after the closure of the present study, a position statement was published on Women's Cardiovascular Health with recommendations of aspects related to all lifestyle pillars as a preventive measure for cardiovascular diseases.^81^It is expected to be a trend, and thus a good attitude for other guidelines to trigger the incorporation of other pillars, as was done in the 2019 Cardiovascular Prevention Guideline, which fits cardiovascular diseases in a generic way.

### Limitations

Among the limitations, the studies gathered for analysis, which serve as a basis for demonstrating the effects of the lifestyle pillars on disease management, are not necessarily randomized clinical trials, due mainly to the ethical reasons already discussed above. The search for articles related to lifestyle and the subjects of the studied guidelines did not meet the rigors of a systematic review, since it was not within the scope of this article.

Furthermore, this study focused on Brazilian medical guidelines, not extending the analysis and comparison to American or European guidelines. Although NCDs are a global problem, the national sphere was chosen first to raise awareness.

### Future steps

When studying lifestyle and its impact on health processes, little is said about environmental and socioeconomic aspects. The Cardiovascular Prevention Guideline published in 2019, addressed in this study, presents a section on the impact of these factors on health care, which is a new line of study to be explored that may even fit as a future lifestyle pillar not related to behavioral measures.

In addition, another possible way to improve this study is to report on the NCDs that cause the most deaths in Brazil, not only considering the ICD-10 category informed by the SIM.

## Conclusion

Rarely are the six pillars of lifestyle contemplated in Brazilian guidelines for AMI, DM, and COPD. The literature review identified evidence of all lifestyle pillars to offer a holistic approach for the management and prevention of these NCDs.

## * Supplemental Materials

For additional information, please click here.
